# 

**Published:** 1916-05

**Authors:** 


### THE SOUTHERN MEDICAL ASSOCIATION.

The next meeting of the Association will be held at Atlanta in November. This statement should be of peculiar interest to all Georgia physicians if for no other reason than that of State pride. At the last meeting in Dallas, the Texans did wonderful things in the matter of membership. The leaders had promised the year before that if the meeting came to Dallas, they would have a certain number of new members to show the appreciation of the Association in the minds of the Texans. They made good and hundreds of new members are today enjoying the benefits of the Association.

The exact number of members is not given because Texas and Georgia differ in population and size in such a way that percentages rather than concrete amounts are needed for comparison. It is true that Georgians went into the Association freely at first and already have a large representation there. It is also true that there is a large number of men who have not seen fit to join. To both of these groups this appeal is made.

The Southern Medical Association has grown up before you and you know what it is. It started with meetings among neighboring states and soon grew to be so valuable to its members and to its guests that its field was extended and it naturally embraced all the Southern States. Nothing of a propaganda nature placed it where it is—the third largest medical society in the world. It grew because of its inherent qualities that proved themselves valuable to the people it reached. It concerns itself primarily with just those things that the Southern practitioner wants to know and it does this without any taint of sectionalism. It is no more sectional in its ideas than it is in its study of pellagra or malaria or hook worm or blackwater fever. These diseases are here in the South more than elsewhere and they are studied for their local and their world-wide importance by minds cultivated to investigate and to determine great things anywhere in the world. The limitation of the membership is for convenience and to give greater opportunity to the men who do similar work, for consultation and personal touch.

The advantages are here but hinted at. They are increased through the publication of one of the best Medical Journals in the world that brings to every member the work of the Association as well as the comment and critical analysis of the editorial staff upon the advances in the world at large.

To each member in Georgia we send a greeting and with it the request that he finds his confreres who are not members and urges them to send their applications either to Dr. M. L. Boyd, Candler Building, Atlanta, Ga., or to Dr. Seal Harris, Birmingham, Ala. Dr. Boyd is Chairman of the Committee on Membership for the next meeting. Dr. Harris is as everyone knows, the Secretary of the Association.

To every man who is not a member, we urge that he become one and assist in making the Association the more influential by his membership. There are great things to be done in prophylactic medicine alone and these can be accomplished by unified work where there is no loss of motion. There are legislative matters to be handled and these will have proper issue when the legislators realize that the Profession as a whole is speaking through the association and that there is no "politics" in the laws proposed.

In addition to all these, there is camaraderie amongst the members that has a fine influence in stimulating each to better work and a greater cheerfulness in life.

The only necessary preliminary is to be a member of one's County Society. If there should be no such Society, write to Dr. Boyd for further and particular information.

Let us get together and show that in Georgia there are no eligible men who are not members of the Association.

To our other readers who may think that this appeal is insular, we have to say that Georgia needs it and that if any State wishes to enter into competition with us in the matter of membership, we should be delighted to have them put themselves on record with Dr. Harris and go to it as fast as they can. It is the kind of contest that can not but do all of us good.

---

### ANNUAL MEETING OF THE AMERICAN MEDICAL EDITORS' ASSOCIATION.

The annual meeting of this Association will meet at the McAlpin Hotel, New York City, on October 25th and 26th.

A most interesting programme is in course of preparation and the Local Committee composed of the following members is an assurance of a successful convention:

Dr. Thomas L. Stedman, Editor Medical Record, Chairman.

Dr. R. H. Sayre, New York Medical Journal.

Dr. Brooks H. Wells, Editor Amer. Jour. of Obstetrics.

Dr. Frank C. Lewis, International Jour. of Surgery.

Dr. Ira S. Wile, American Medicine.

The officers for the Association for 1915 and 1916 are as follows:

Dr. Edward C. Register, President. (Charlotte Medical Journal, Charlotte, N. C.)

Dr. W. A. Jones, 1st Vice-President. (Journal Lancet, Minneapolis, Minn.)

Dr. G. M. Piersol, 2nd Vice-President. (Amer. Journal Medical Sciences, Philadelphia, Pa.)

Dr. J. McDonald, Jr., Secretary and Treasurer. (American Journal of Surgery, New York.)

Executive Committee:

Dr. C. F. Taylor, (Medical World, Philadelphia, Pa.)

Dr. John C. MacEvitt, (N. Y State Journal of Medicine, New York.)

Dr. A. S. Burdick, (Amer. Journal of Clin. Med., Chicago, Illinois.)

Dr. Joseph MacDonald, Jr., (Amer. Journal of Surgery, New York.)

The meeting on October 25th and 26th will be devoted exclusively to problems of a strictly journalistic nature, which will be of importance and interest to every editor and publisher of a medical journal. Among the papers to be presented are the following:

"Editorial Control," "The Editor5s Prerogative in Editing Original Articles," "Book Reviews in Medical Journal," "Problems of the Subscription Department," "The Relationship Between Medical Journals of the Day," "The Up-Lift in Medical Journalism," 'The Influence of the Medical Press and Profession in Public Affairs," "The Rights of an Author in the Disposition of his Contribution," etc.

### OUR NEW SUBSCRIBERS

Drs. Terrell & Clark--------Ga.
Jas. Turner ---------- W. Va.
L. W. Kornegay---------N. C.
Jno. D. Hanson------------La.
J. S Rhanu--------------S. C.
A. J. Jervey------------ S. C.
E. L. Angier-------------Tex.
W. M. Fust------------N. C.
D. D. Hatfield---------W. Va.
Dr. E. B. Morgan---------Tex.
Dr. R. H. Bryant---------N. V.
Dr. H. C. Slaughter----W. Va.
Dr. C. S. Sherrell--------N. C.
Dr. C. S. Howell--------S. C.
Dr. B. M. Montgomery--S. C.
Dr. I. A. Macon--------S. C.
Dr. W. J. Crossland------S. C.
Dr. J. A. Norton--------S. C.
Dr. J. S. Jacoby--------Okla.
Dr. F. S. Robertson----W. Va.
Dr. E. T. Kelly ----------
Dr. A. H. Gregg-------W. Va.
Dr. S. R. Lucas----------S. C.
Dr. F. K. Rhodes--------S. C.
Dr. E. C. Ferguson------Texas
Dr. H. M. Batson------ W. Va.
Dr. A. C. Owings--------S. C.
Dr. C. F. Ross----------S. C.

Dr. C. L. Hunsucker------N. C.
Dr. J. L. Anderson-------S. C.
Dr. T. G. Sharpe-----------S. C.
Dr. R. H. McFaden------S. C.
Dr. J. H. Hunter--------S. C.
Dr. V. M. Long----------N. C.
Dr. Robt. Wriston---- W. Va.
Dr. S. W. Rankin--------N. C.
Dr. J. L. Whitehead-----N. C.
Dr. H. C. McDowell------S C.
Dr. D. S. Porter--------S. C.
Dr. P. K. Swintzer--------S. C.
Dr. R. W. Timberlake-W. Va.
Dr. E. H. McClendon------Ga.
Dr. S. A. Hardman----W. Va.
Dr. W. P. Herbert------N. C.
Dr. H. F. Lawson------W. Va.
Drs. Bradford & Sharpe--S. C.
Dr. R. W. Fisher------W. Va.
Dr. S. P. Shirkey------W. Va.
Dr. W. N. Haynes------W. Va.
Dr. J. R. Younmans--------Ga.
Dr. D. L. Yost--------W. Va.
Dr. J. E. Mahoney------Okla.
Dr. B. D. Tyler-----------Ky.
Dr. R. L. Cockfield-------S. C.
Dr. Walter Wright------Okla.
Dr. F. L. Carpenter----S. C.
Dr. G. T. Grisinger----W. Va.
Dr. S. M Browne---------S. C.
Dr. G. C. Cole-------------Ga
Dr. E. S. Colvin-----------Ga.
Dr. S. L. Katzoff----------Ga.
Dr. H. W. Watson---------Ga.
Dr. J. J. Martin----------Ga.
Dr. J. Carothers----------Ga.
Dr. O. H. Morris----------Ga.
Dr. J. C. Oakshette--------Ga.
Dr. H. M. Luning----------Ga.
Dr. L. M. Hill-------------Ga.
Dr. Thos. Slater----------Ga.
Dr. Geo. S. Morse----------Ga.
Dr. I. A. Dailey----------Ga.
Dr. Geo. Carefoot---------Ga.
Dr. M. W. McLarty------Ga.
Dr. D. B. Tallant----------Ga.
Dr. O. T. Malone----------Ga.
Dr. H. J. Wright----------Ga.
Dr. H. E. Duffy------------Ga.
Dr. M. R. Gibson---------N. C.
Dr. J. G. Raby----------N. C.
Dr. J. L. Duckett--------S. C.
Dr. D. A. Garrison------N. C.
Dr. H. S. Keister------W. Va.
Dr. V. F. Couch----------N. C.
Dr. T. C. Maguire---------Fla.
Dr. H. D. Wood-----------Ark.
Dr. Vance W. Brabham--S. C.
Dr. L. Abrasom-------------La.
Dr. J. M. Crawford------N. C.
Dr. C. H. Maxwell----W. Va.
Dr. O. P. Nuckols---------Ky.
Dr. George A. Griffith--N. C.
Dr. C. I. Wall----------W. Va.
Dr. Thel. Hooks--------N. C.
Dr. Roger Coke---------Texas
Dr. M. C. Scott-----------Va.
Drs. Newton & Richer--Texas
Dr. Robert Wilson------S. C.
Dr. William L. Dunn----N. C.
Dr. D. A. Barow--------S. C.
Dr. T. H. Marble-------Tenn.
Dr. Robert Riston------W. Va.
Dr. . B. Backus------W. Va.
Dr. J. E. Cannaday----W. Va.
Dr. W. S. Beane-------W. Va.
Dr. L. C. Stokes--------S. C.
Dr. A. M. Showalter------Va.
Dr. R. Moreland-----------Ky.
Dr. Wm. H. Wadsworth-N. C.
Dr. R. H. Walker----W. Va.
Dr. T. G. Gantt------------Ga.
Dr. W. H. Morgan---------Ala.
Dr. H. C. Martin------W. Va.
Dr. M. L. Brown--------Texas
Dr. John A. Gibson------Va.
Dr. R. M. Walker------Texas
Dr. E. O. Powers----------La.
Dr. W. W. Harloe------W. Va.
Dr. Chas. O. Hooks----Texas
Dr. G. H. Fitchgerald---Ariz.
Dr. Dallas Southard----Texas
Dr. Francis Ridley----W. Va.
Dr. J. W. Kenney------Texas
Dr. F. M. Wilson--------Texas
Dr. E. L. Howard------Texas
Dr. J. G. Rogers--------S. C.
Dr. C. A. Dexter----------Ga.
Dr. T. Adair----------W. Va.
Dr. Vance Rawson---------Ky.
Dr. A. D. Simmington-----Ala.
Dr. W. B. Steavens----W. Va.
Dr. J. L. McCarty------W. Va.
Dr. J. T. Longest---------Ark.
Dr. J. O. Matthews----Texas
Dr. J. C. Van Nuys----Texas
Dr. J. A. Williams------N. C.
Dr. L. F. Carlton---------Fla.
Dr. H. G. Stoneham------Va.
Dr. A. A. Williams--------Ga.

Dr. W. W. Conyer----W. Va.
Dr. Tilman Ramsey-------Ky.
Dr. A. J. Buist----------S. C.
Dr. L. O. Schwartz----W. Va.
H. P. B. Bonner--------N. C.
O. E. Clements--------Texas
L. M. Ellis------------Texas
H. H. Edmondson------Tenn.
Dr. Jas. R. Middlebrooks-Tex.
Dr. C. Curtice Collins---Fla.
Dr. S. E. Johnson--------Fla.
Dr. L. A. Peek----------Fla.
Dr. H. C. Galey----------Fla.
Dr. J. A. Stanford--------Fla.
Dr. S. L. Cheshire--------Ga.
Dr. J. G. Devane----------Ga.
Drs. Woodward & Hall----Ga.
Dr. Cecil Garrenton-----N. C.
Dr. Jno. L. Lane------N. C.
Dr. G. F. McInnes------S. C.
Dr. Geo. Y. Massenburg---Ga.
Dr. Wm. A. Reimer----W. Va.
Dr. Ben F. Royal------N. C.
Dr. Joseph H. Saunders--N. C.
Dr. W. A. Carr--------W. Va.
Dr. M. M. Edwards------S. C.
Dr. D. D. Hatfield------W. Va.
Dr. Wm. F. Ferguson--W. Va.
Dr. V. L. Wetherby----W. Va.
Dr. G. W. Smith------W. Va.
Dr. G. C. Balke--------W. Va.
Dr. J. W. Wilkin------N. C.
Dr. Geo. H. Gale--------Ohio
Dr. Geo. Fordham----W. Va.
Dr. R. L. Foscer-------W. Va.
Dr. D. G. Preston------W. Va.
Dr. A. U. Tieche-------W. Va.
Dr. Thos. C. Thompson----Fla.
Dr. H. R. Drew----------Fla.
Dr. J. C. Dickerson-------Fla.
Dr. S. S. Sission----------Fla.
Dr. E. W. Brenham-----Texas
Dr. W. D. Chappell------S. C.
Dr. C. C. Stephenson----S. C.
Dr. S. W. Bull--------W. Va.
Dr. T. H. Bryan------W. Va.
Dr. E. Lartigue----------Fla.
Dr. J. A. Dye---------W. Va.
Dr. L. M. Daniels------S. C.
Dr. W. C. Boylston----S. C.
Dr. Edgar Peters---------Fla.
Dr. F. B. Johnson--------S. C.
Dr. J. E. Marschner----W. Va.
Dr. J. C. Mathews------S. C.
Dr. L. L. Highsmith--W. Va.
Dr. G. M. Sturgell--------Ky.

